# Pediatric atopic dermatitis: biomarker advances in severity, persistence, and non-invasive monitoring

**DOI:** 10.3389/fped.2026.1902799

**Published:** 2026-08-18

**Authors:** Zhuoyuan Li, Yuanjun Li

**Affiliations:** 1 School of Clinical Medicine, Guizhou Medical University, Guiyang, China; 2 Department of Dermatology, Children’s Hospital of Shanxi Province, Taiyuan, China

**Keywords:** atopic dermatitis, biomarkers, children, detection techniques, endotype, personalized treatment

## Abstract

Atopic dermatitis (AD) in children differs immunologically and in barrier characteristics from adult AD, yet current severity assessment still relies heavily on clinical scoring systems with inherent subjectivity. This review summarizes advances in biomarkers associated with disease severity and persistence, as well as non-invasive detection techniques and targeted therapies. TARC/CCL17, periostin, total IgE, and eosinophil counts show consistent utility in grading severity, whereas FLG loss-of-function mutations, VEGF, S100A8/A9, and early skin barrier indicators are promising predictors of disease persistence and atopic march progression. Tape stripping and sebum RNA analysis enable safe, repeated monitoring in children. Despite these advances, most biomarkers still lack pediatric-specific cut-offs and large-scale validation. Future efforts should focus on establishing multi-marker panels, pediatric reference intervals, and machine learning models for endotype classification. Incorporating these tools into routine practice would facilitate early risk stratification, precise targeted treatment selection, and ultimately more personalized management of pediatric AD.

## 1 Introduction

Atopic dermatitis (AD) is a chronic relapsing inflammatory skin disease characterized by inflammation, skin lesions, and pruritus (1, 2). Globally, AD affects as many as 20%–25% of children and 10% of adults, and nearly half of all cases (approximately 45%) present within the first 6 months of life (1, 2).

AD is highly heterogeneous, and significant differences in treatment response to the same therapy are observed among patients (3). In recent years, the introduction of novel targeted therapies has driven the need for patient stratification based on biomarkers. Currently, the management of AD patients primarily relies on clinical scoring systems such as the SCORing Atopic Dermatitis (SCORAD) index and the Eczema Area and Severity Index (EASI) to assess disease severity. However, these scoring systems are susceptible to bias from patients' subjective perceptions and variations in physicians' interpretation, and they fail to account for individualized pathogenesis or differentiate patients' endotype characteristics. Global trials have shown that dupilumab (based primarily on adult data) enables only 44%–65% and 30%–51% of patients to achieve ≥75% improvement in EASI (EASI-75) and≥90% improvement in EASI (EASI-90), respectively, highlighting the substantial variability in treatment response and the urgent need for endotype-driven therapeutic strategies (4). In the pediatric population, dupilumab is currently the only biologic approved for moderate-to-severe AD in children as young as 6 months of age. Real-world data from children aged 6 months to 5 years with moderate-to-severe AD demonstrated a mean EASI reduction of 89.7% at week 52, with EASI-75 and EASI-90 response rates of 79.5% and 59.0%, respectively, and no negative impact on growth (5); Long-term extension data (up to 104 weeks) further support its favorable long-term safety and sustained efficacy in this age group (6). Nevertheless, a considerable proportion of patients still do not achieve EASI-90, suggesting that endotype-guided treatment optimization remains relevant even in the pediatric population.

The “atopic march” refers to the progressive process beginning with infantile eczema and subsequently developing into asthma, allergic rhinitis, and food allergy, with AD generally considered the initial manifestation of the atopic march (1, 4). Evidence from epidemiological and animal studies points to a link between AD and later-onset allergic airway inflammation, with skin barrier impairment and cutaneous inflammation potentially facilitating allergen sensitization (1). Among these, filaggrin (FLG) loss-of-function mutations can increase the risk of multiple comorbid conditions, and succinate and mitochondrial DNA also play a role in the progression from AD to food allergy and intestinal inflammation. These findings suggest that AD might serve as a “gateway” to multiple comorbidities (7, 8). However, not all children with AD will develop other conditions along the atopic march; approximately 50% of pediatric patients do not experience such disease progression (1).

In children with AD, a Th2-dominated immune response predominates early in life, accompanied by significantly upregulated Th9 and Th17 responses, whereas adults exhibit a higher Th22 response (1, 4). The AD phenotype in Asian patients displays a mixed feature of AD and psoriasis, characterized by upregulation of Th17 and Th22, as well as lower Th1 expression compared to European and American patients (2, 4, 9). These age- and ethnicity-related immunological differences highlight the special significance of biomarker research focusing on pediatric AD.

Recent reviews have comprehensively summarized biomarkers in atopic dermatitis. This review uniquely focuses on predictors of disease persistence, non-invasive monitoring techniques, and early risk stratification for the atopic march.

Therefore, this article systematically summarizes biomarkers associated with disease severity and persistence in pediatric atopic dermatitis, along with advances in non-invasive detection techniques and targeted therapies. By clarifying the clinical value and limitations of various biomarkers, this article aims to provide clinicians with a practical basis for early risk stratification, dynamic disease monitoring, and endotype-driven personalized treatment of pediatric AD, thereby improving long-term outcomes for affected children.

## 2 Pathological basis for AD

### 2.1 Skin barrier

#### 2.1.1 Filaggrin(FLG)

FLG loss-of-function mutations are considered the strongest genetic risk factor associated with the development of AD (1, 2, 10, 11). FLG deficiency can lead to altered skin pH, impaired lipid synthesis, reduced natural moisturizing factor (NMF), and increased transepidermal water loss (TEWL) (2, 11). Notably, the downregulation of FLG exhibits age-related differences; it is most pronounced in adult AD patients and less evident in pediatric AD (12). Th2 cytokines IL−4,IL−13,IL−22,IL−31 can directly downregulate FLG expression (2, 13).

#### 2.1.2 Claudin-1 (CLDN1)

Gruber et al. found that CLDN1 expression was significantly downregulated in lesional AD skin, but no significant difference was observed between non-lesional AD skin and healthy controls (14). However, a review by Katsarou et al. indicated that CLDN1 can be downregulated in both lesional and non-lesional skin, a discrepancy potentially attributable to differences in study populations and detection methods (15). Inflammation (particularly Th2 inflammation) and scratching can reduce CLDN1 expression (15).

Epidermal barrier disruption caused by scratching, microbial adhesion, and chemical irritation stimulates keratinocytes to release alarmins, including thymic stromal lymphopoietin (TSLP), Interleukin-25 (IL-25), and IL-33, which activate Th2 immune responses. Th2 cytokines further downregulate the expression of barrier protein genes such as FLG, establishing a vicious positive feedback loop (11, 16).

### 2.2 Th2 inflammation

Th2 cells are a crucial subset of CD4+ helper T cells that play a key role in type 2 immunity by secreting cytokines such as IL-4, IL-5, IL-9, IL-13, and IL-31 (16).

#### 2.2.1 Alarmin release triggered by barrier disruption

Following epidermal barrier disruption by various stimuli, keratinocytes release alarmins TSLP, IL-25, and IL-33 (11, 16). TSLP binds to receptors on dendritic cells, thereby promoting the differentiation of naïve T cells toward Th2 cells (16). Keratinocyte-derived IL-33 can activate group 2 innate lymphoid cells (ILC2s) to produce IL-5 and IL-13, inducing eosinophil accumulation (16).

#### 2.2.2 IL-4 and IL-13 as core effector molecules

IL-4 and IL-13 are the two most central Th2 effector molecules in the pathogenesis of AD (2, 17). They not only promote IgE production by B cells and the activation and recruitment of eosinophils but also downregulate the expression of FLG, keratins, involucrin, and lipids (2, 13, 16, 18). IL-4 and IL-31 also directly stimulate pruriceptive sensory neurons, leading to neurogenic pruritus (2, 16). IL-13 expression is significantly upregulated in acute and chronic lesional skin as well as non-lesional skin and is closely associated with elevated eosinophil counts and AD severity (19). Studies have shown that reduced IL-13 levels correlate with treatment response and improved clinical outcomes (19, 20).

#### 2.2.3 IL-31 as a core mediator of pruritus

Pruritus is the main symptom of pediatric AD, and IL-31 is closely associated with the induction of pruritus (21). IL-31 is primarily produced by activated Th2 cells, and its receptor is expressed on keratinocytes and dorsal root ganglion neurons (21, 22). IL-31 not only directly induces pruritus but also leads to basal cell proliferation and impaired barrier function (21).

### 2.3 Th17/Th22 inflammation

In addition to the Th2 pathway, Th17, Th22, and Th9 are involved in the pathogenesis of AD, with significant differences observed between children and adults, and between Asian and European/American populations (1, 3, 4, 9).

#### 2.3.1 Th9 and Th17 predominance in children, Th22 predominance in adults

Compared to adults, pediatric AD patients show higher induction levels of Th17- and Th9-related cytokines (1, 3). Skin samples from early-onset pediatric AD demonstrate significantly upregulated Th9 and Th17 responses, whereas adult AD is characterized by Th22 inflammation (1, 4).

#### 2.3.2 Role of IL-22

IL-22 is the signature cytokine of Th22 cells and is upregulated in both acute and chronic AD (2). IL-22 disrupts the skin barrier by inhibiting the expression of epidermal differentiation proteins, enhances antigen sensitization, promotes Th2 inflammation, and participates in the pathogenesis of chronic pruritus (2).

### 2.4 Microbiota

Significant dysbiosis of the skin microbiota exists in AD patients, primarily manifesting as reduced microbial diversity and an increase in opportunistic pathogens (1, 2, 23).

#### 2.4.1 Staphylococcus aureus as a core pathogen

The colonization rate of Staphylococcus aureus is only 15%–30% in the general population, but the detection rate in lesional skin of children with AD can reach 70%–90% (24–26). Toxins produced by S. aureus can act as superantigens, penetrating the compromised epidermis and triggering dermal dendritic cells to drive Th2 cell expansion (27). S. aureus can also induce IgE production by B cells, keratinocyte apoptosis, and mast cell degranulation (24). Furthermore, S. aureus colonization can trigger IL-36*α* expression, which in turn induces the pro-inflammatory cytokine IL-17 (25, 26).

#### 2.4.2 Malassezia and other microorganisms

The colonization rate of Malassezia species in AD patients can reach 100%, and in children with AD, the colonization counts of Malassezia dermatis and Malassezia sympodialis are higher than in healthy individuals (2, 24). The diversity of the oral, skin, and gut microbiota is significantly reduced in children with AD (23). Skin microbiota dysbiosis, characterized by the predominance of S. aureus and Malassezia, is a key factor influencing disease progression (1).

**Figure 1:**
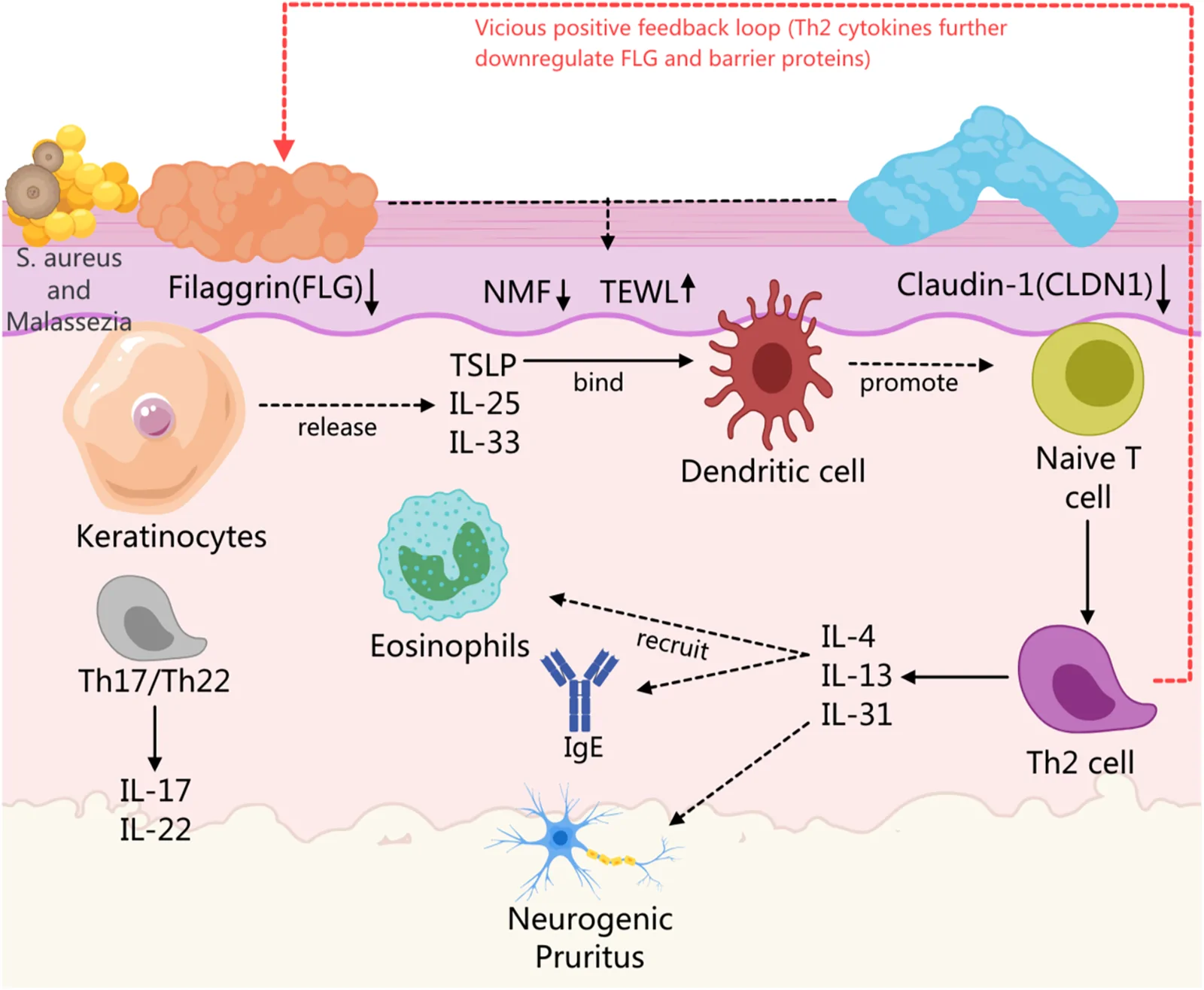
Schematic illustration of the pathogenesis of atopic dermatitis. The figure shows skin barrier disruption (decreased FLG, claudin-1, and NMF; increased TEWL), release of alarmins TSLP, IL−25, IL−33 from keratinocytes, activation of dendritic cells and differentiation of naive T cells into Th2 cells, production of IL-4, IL-13, and IL-31 by Th2 cells (leading to IgE production, eosinophil recruitment, and neurogenic pruritus), production of IL-17 and IL-22 by Th17/Th22 cells, colonization of Staphylococcus aureus and Malassezia on the skin surface, and a positive feedback loop where Th2 cytokines further downregulate FLG and other barrier proteins. This figure was created with MedPeer (medpeer.cn).

The key pathological mechanisms of atopic dermatitis, including skin barrier disruption, Th2 inflammation, and microbial dysbiosis, are summarized in Figure 1.

## 3 Biomarkers associated with severity

The identification, validation, and clinical translation of biomarkers is a complex and time-consuming process. Despite considerable research efforts in AD, no biomarker has yet been implemented in routine clinical practice. Obtaining detailed molecular data from sufficiently large patient cohorts is crucial for stratified analysis and advancing biomarker research (28).

### 3.1 Thymus and activation-regulated chemokine (TARC/CCL17)

TARC/CCL17 is a Th2 chemokine that binds to the CCR4 receptor and recruits Th2 cells to sites of inflammation (16). TARC has been identified as the most reliable and efficient biomarker to date (1, 4).

Linear regression analysis shows that TARC is positively correlated with objective severity (EASI), patient-reported symptoms (POEM), and pruritus Numerical Rating Scale (NRS) scores (29). A meta-analysis further confirmed that the pooled correlation coefficient between TARC and AD severity was 0.6 in longitudinal randomized trials and 0.64 in cross-sectional studies, indicating that TARC can consistently reflect disease severity across different study designs (4). Despite these consistent correlations, the lack of pediatric-specific cut-off values limits the direct clinical applicability of TARC in routine practice, highlighting the necessity of establishing age-stratified reference intervals in future studies to better utilize this reliable biomarker.

### 3.2 IgE and eosinophils

IgE and eosinophils are classic type 2 immunity-related biomarkers in AD, both regulated by Th2 cytokines. Combining IgE levels and eosinophil counts can help differentiate extrinsic AD (elevated IgE, often with increased eosinophils) from intrinsic AD (normal IgE, usually with normal eosinophils) (1, 9). However, this dichotomous classification also reveals an inherent limitation of IgE and eosinophils as biomarkers—in children with intrinsic AD, which is not uncommon in clinical practice, the sensitivity of both markers is significantly reduced, limiting their utility as universal severity indicators.

#### 3.2.1 Clinical value and limitations of IgE

Total IgE is usually elevated in extrinsic AD, but its correlation with disease severity is weak and inconsistent (1). IgE is elevated not only in AD but also in other atopic conditions such as asthma and allergic rhinitis, thus exhibiting poor specificity (3). In addition, autoreactive IgE may be associated with atopic comorbidities; AD patients with comorbid type 2 conditions have higher levels of autoreactive IgE (4). Malassezia-specific IgE may also be a potential severity biomarker (4).

#### 3.2.2 Clinical value of eosinophils

Peripheral blood eosinophil counts show a significant positive correlation with EASI, POEM, and pruritus NRS scores (29). Möbus et al. categorized AD patients into eosinophil-high and eosinophil-low subtypes; the eosinophil-high subtype exhibited more pronounced global transcriptomic dysregulation, and disease severity was positively correlated with IL-5 markers (3, 4). AD severity and eosinophilia are the most important factors in combined biomarker profiles (19).

### 3.3 Periostin and DPP-4

Periostin and DPP-4 are both downstream molecules of IL-13, but their clinical evidence profiles are not aligned. Periostin has accumulated supportive data in severity assessment and treatment response prediction, whereas the clinical utility of DPP-4 remains controversial (19).

#### 3.3.1 Clinical value of periostin

Linear regression analysis revealed a significant positive correlation between serum periostin levels and both EASI and pruritus NRS scores (29). Periostin can reflect Th2 inflammation, but its predictive value for disease severity still requires further validation (1). In clinical studies, AD patients with high baseline periostin levels responded better to IL-13 inhibitors (e.g., tralokinumab), suggesting its potential as a predictive marker of treatment response (3, 20).

#### 3.3.2 Clinical value of DPP-4

Although DPP-4 is considered a biomarker of IL-13 activity, current evidence does not support its use as a biomarker for assessing AD severity (19). Maintz et al., in a cohort of 373 AD patients, reported that DPP-4-high patients displayed a clinical phenotype with no clear association with IL-13 or severity scores, and explicitly stated that their findings do not support DPP-4 as a predictive biomarker for AD severity (19). Other studies also failed to find a significant association between serum DPP-4 levels and AD severity (19).

Notably, while Maintz et al. listed DPP-4 alongside periostin as an IL-13-upregulated marker in their introduction, their subsequent analyses revealed that DPP-4 exhibited effects in the opposite direction to IL-13 and periostin (19). This apparent contradiction suggests that DPP-4 expression may be regulated by factors beyond IL-13, and its reliability as a surrogate marker of IL-13 activity warrants further investigation.

### 3.4 CCL26/eotaxin-3, SCCA2, and LDH

CCL26 and SCCA2 are emerging severity biomarkers in AD. Both are downstream molecules of IL-4 and IL-13, elevated in the blood of both pediatric and adult AD patients, and increase with AD severity, serving as surrogate biomarkers for IL-4 and IL-13 activity (29).

Linear regression analysis demonstrated that CCL26, SCCA2, and lactate dehydrogenase (LDH) were all significantly and positively correlated with EASI (29). ROC curve analysis further confirmed that CCL26 and SCCA2 are the best markers for assessing EASI severity, while LDH is the best marker for assessing POEM and pruritus NRS (29). These findings suggest that CCL26, SCCA2, and LDH have promising potential for severity assessment in AD, and further exploration in future studies—particularly in pediatric AD populations—is warranted to evaluate their clinical applicability.

### 3.5 IL-36

IL-36 is the newest member of the IL-1 cytokine family, comprising four subtypes: the pro-inflammatory IL-36*α*, IL-36*β*, IL-36*γ*, and the anti-inflammatory IL-36 receptor antagonist (IL-36Ra). An imbalance between pro-inflammatory and anti-inflammatory subtypes can lead to inflammatory responses (26). IL-36 is associated with neutrophilic inflammation and disease severity in AD and represents a potential marker for evaluating clinical improvement of skin lesions in clinical trials (1, 25).

However, the expression pattern of IL-36 in AD is not consistent across studies. Tsang et al. and Ahmad et al. both reported elevated mRNA expression of IL-36*α* and IL-36*γ* in lesional skin of moderate-to-severe AD patients compared with non-lesional skin, which was also confirmed by tape-stripping studies (25, 26). In contrast, Grolleau et al. using immunohistochemistry, failed to detect IL-36*α* and IL-36*γ* protein expression in AD lesional skin (30).

This mRNA-protein discordance may be attributed to several factors. First, IL-36 isoforms require N-terminal proteolytic cleavage for optimal biological activity, and their protein levels are regulated by post-transcriptional mechanisms, translational efficiency, and protease activity—thus, increased mRNA does not necessarily translate into elevated protein levels. Second, the methodological approaches differ across studies: Tsang et al. and Ahmad et al. employed RNA sequencing and RT-PCR (highly sensitive for mRNA), whereas Grolleau et al. used immunohistochemistry (which is influenced by antibody specificity and antigen retrieval conditions). Third, IL-36 expression may vary across different skin layers, and the sample sources and processing methods were not identical across studies.

Notably, even the difference between lesional and non-lesional skin is not consistently observed across studies—some found elevated expression only in lesional skin, while others also detected differences in non-lesional skin. This inconsistency and methodological dependence suggest that IL-36, at this stage, is not yet a reliable first-line severity biomarker for AD, and its clinical applicability requires further evaluation under standardized detection protocols.

### 3.6 IL-8

Using the tape-stripping method, it was found that during topical corticosteroid treatment, stratum corneum IL-8 (scIL-8) levels in the forearm and abdomen were significantly downregulated after treatment compared to pre-treatment levels, and the degree of reduction correlated with the improvement of skin symptoms (31). Moreover, scIL-8 levels in the forearm and abdomen were significantly and positively correlated with TEWL and TARC levels, suggesting that scIL-8 levels are associated with the severity of local skin inflammation in AD (31).

### 3.7 MicroRNA-155 (miR-155)

miR-155 is significantly elevated in the serum of children with AD, but it shows no statistical correlation with SCORAD, eosinophils, total IgE, or Th2 cytokines, suggesting that it may primarily serve as a diagnostic biomarker rather than a severity marker (32). However, given the small sample size of the study (12 AD patients and 9 healthy controls), the value of miR-155 as a diagnostic biomarker requires further validation in larger cohorts.

In summary, the currently studied severity biomarkers for AD can be broadly categorized into several groups: TARC/CCL17 has been consistently validated as the most reliable severity marker across multiple studies; periostin has shown correlations with EASI and pruritus scores and demonstrated potential in predicting treatment response, but its independent predictive value requires further validation; eosinophil counts and total IgE have clinical utility in specific contexts (e.g., distinguishing extrinsic from intrinsic AD), but eosinophils have limited sensitivity in intrinsic AD, while IgE, in addition to this issue, also has poor specificity and weak correlation with severity; emerging markers such as CCL26, SCCA2, and LDH show promise but require large-scale validation; IL-36 has preliminary evidence supporting its association with disease severity, but the discordance between mRNA and protein levels warrants further investigation; IL-8 has been shown in tape-stripping studies to correlate with local inflammation severity and treatment response, but its evidence as an independent severity marker remains limited; miR-155 has insufficient evidence to support its use as a severity marker; and DPP-4 is not supported by current evidence for AD severity assessment.

The biomarkers associated with disease severity in pediatric atopic dermatitis, along with their sample sources, correlation strength, and special considerations in children, are summarized in Table 1

**Table 1:** Biomarkers associated with disease severity in pediatric atopic dermatitis.

Biomarker	Clinical utility	Clinical status	Key limitation/Note	References
TARC/CCL17	Most reliable severity marker	Validated	Pediatric cut-offs not established	(1, 4, 16, 29)
Total IgE	Combined with eosinophil count to distinguish extrinsic vs. intrinsic AD	Validated (with limitations)	Poor specificity; weak correlation with severity; limited sensitivity in intrinsic AD	(1, 3, 4)
Eosinophil count	Combined with IgE to distinguish extrinsic vs. intrinsic AD	Validated (with limitations)	Limited sensitivity in intrinsic AD	(3, 4, 19, 29)
Periostin	Predicts response to IL-13 inhibitors	Promising	Independent predictive value for severity requires further validation	(1, 3, 19, 29)
CCL26	Best marker for EASI severity	Emerging	Requires large-scale validation	(29)
SCCA2	Best marker for EASI severity	Emerging	Requires large-scale validation	(29)
LDH	Best marker for POEM and pruritus severity	Emerging	Requires large-scale validation	(29)
IL-36*α*/*γ*	Elevated in moderate-to-severe AD	Controversial/Exploratory	mRNA-protein discordance; inconsistent across studies	(1, 25, 26, 30)
IL-8 (scIL-8)	Monitors local treatment response	Evidence limited	Local inflammation marker; not established as independent severity marker	(31)
DPP-4	None	Not supported	Evidence does not support use for severity assessment	(19)
miR-155	Elevated in pediatric AD	Exploratory	No correlation with severity; may serve as diagnostic rather than severity marker	(32)

## 4 Biomarkers associated with disease persistence prediction

### 4.1 FLG mutations

FLG loss-of-function mutations are closely associated with early onset, severity, and persistence of AD, and increase susceptibility to food allergy and the atopic march (1, 33). FLG mutations can increase the risk of multiple comorbidities by 2- to 3-fold (7), and the odds ratio (OR) for the association between FLG loss-of-function mutations and elevated AD risk is 3.39 (95% CI 2.73–4.23) (13).

FLG loss-of-function mutations are also risk factors for asthma, food allergy, and rhinitis. The hazard ratio for developing asthma in the context of eczema with FLG mutations is 3.16 (13). FLG mutations can also increase the risk of allergy to multiple foods, such as milk, eggs, and fish, independent of eczema (13). The association between FLG loss-of-function mutations and IgE-mediated peanut allergy has been validated in three populations: Canada, the Netherlands, and the United Kingdom (13). Therefore, FLG mutation testing may help identify high-risk children and provide a basis for early intervention and prevention of comorbidities.

### 4.2 VEGF

Low serum VEGF levels are associated with the persistence of pediatric AD. Lauffer et al. found that the factors with the greatest impact on disease persistence included SCORAD score at 3 years of age, trigger factors, and low VEGF levels, with a sensitivity of 81.8% and a specificity of 82.4% for predicting disease persistence at 7 years of age (34). Children with higher serum VEGF levels were more likely to achieve disease remission by 7 years of age (34). This suggests that VEGF levels may serve as a predictor of disease outcome in pediatric AD.

### 4.3 S100A8/A9

S100A8/A9 levels can predict the persistence of AD in infants with wild-type FLG (35). Stamatas et al. found that in wild-type FLG infants, skin surface S100A8/A9 levels at 8 weeks of age were closely associated with persistent AD between 6 and 12 months of age (35). Children who had AD at both 6 and 12 months exhibited the highest levels, followed by those with AD only at 6 months, while those without AD at either time point had the lowest levels (35). This progressive pattern was only observed in the FLG wild-type group and not in FLG mutation carriers (35).

Notably, in FLG mutation carriers, none of the biomarkers measured at 8 weeks of age showed significant associations with subsequent AD outcomes (35). This discrepancy suggests that early-onset AD may involve two distinct molecular pathways—one driven by FLG mutation-related barrier defects and the other characterized by early inflammatory activation. S100A8/A9, as an inflammatory marker, primarily reflects the latter pathway. Furthermore, this finding suggests that the predictive value of biomarkers may be subject to time-window and population-specific effects—FLG mutations may already constitute a stronger early risk signal, whereas elevations in skin surface inflammatory markers may primarily reflect non-FLG mutation-driven inflammatory pathways.

### 4.4 Succinate and mtDNA

Clinical studies have shown that the levels of succinate and mitochondrial DNA (mtDNA) in the peripheral plasma of children with AD are significantly higher than those in non-AD children (8). Plasma mtDNA concentration is also positively correlated with EASI score and IgE levels (36).

Animal experiments further demonstrated that succinate mediates the progression of type 2 inflammation from the skin to the small intestine via the tuft cell-ILC2 axis, while mtDNA participates in the atopic march from the skin to the intestine through the STING pathway (8). These findings suggest that succinate and mtDNA may not only serve as key mediators in the progression from AD to food allergy and intestinal inflammation, but also function as early warning biomarkers to help identify children at risk of developing these comorbidities.

### 4.5 Staphylococcus aureus

Early colonization with Staphylococcus aureus is closely linked to the development of AD and its progression to comorbidities. A prospective birth cohort study found that S. aureus colonization in the first few months of life preceded the onset of AD (27). Data from the LEAP study further indicate that infants colonized with S. aureus face a heightened risk of peanut and egg allergy during the first 5 years of life, independent of AD severity (27). These findings suggest that early S. aureus colonization may be associated with the initiation of the atopic march.

### 4.6 TARC

TARC also shows modest early predictive value. A Copenhagen birth cohort study on infant skin showed that skin TARC levels at 2 months of age were significantly higher in children who developed AD within 12 months, but its predictive performance was limited (AUC = 0.64), significantly lower than that of skin lipid markers (phytosphingosine: 75.6% accuracy; a combination of five lipid ratios: 89.4% accuracy) (10, 37).

### 4.7 Barrier-related markers

#### 4.7.1 NMF and TEWL

NMF and TEWL are important indicators for assessing skin barrier function. Reduced NMF levels are usually associated with FLG deficiency mutations and can increase the risk of developing AD (1). High TEWL (>6.5 g/m^2^/h) in the neonatal period indicates skin barrier dysfunction and can increase the risk of developing AD in the first year of life (10). However, TEWL measurements show considerable variability and are influenced by environmental conditions, measurement sites, and procedural factors, and findings across studies have not been consistent (10).

#### 4.7.2 Ceramides (CER) and phytosphingosine (PHS)

Unlike the functional measures above, stratum corneum lipid profiles directly reflect the structural integrity of the skin barrier. Reduced levels of stratum corneum ceramides are a key feature of skin barrier dysfunction in AD. The ratio of sphingoid base chain length of carbon 18 to carbon 20 is the best lipid biomarker for predicting AD (1, 10). Studies have shown that PHS levels at 2 months of age are significantly lower in children who later develop AD, with a predictive accuracy of 75.6% (10, 37).

### 4.8 Multi-factor prediction models

Combinations of multiple skin lipid markers have demonstrated good predictive performance. For example, combinations of different carbon chain lengths of dihydrosphingosine and sphingosine, the ratio of phytosphingosine to sphingosine, and the corresponding ceramide carbon chain ratios can achieve a combined predictive accuracy of 89.4%, outperforming single lipid markers (10, 37).

In summary, the biomarkers discussed in Section 4 differ substantially in their predictive value, target populations, and strength of evidence. FLG mutations have the strongest genetic evidence, increasing the risk of multiple comorbidities by 2- to 3-fold and being closely associated with asthma, food allergy, and the atopic march; VEGF is a component of a combined prediction model and requires combination with clinical factors (SCORAD at 3 years of age, trigger factors) for comprehensive assessment, whereas S100A8/A9 is only effective in FLG wild-type infants; skin lipid markers demonstrate the best predictive performance (89.4% accuracy for a combination of five lipid ratios); barrier function measures (NMF, TEWL) and barrier composition markers (ceramides, phytosphingosine) can help identify early skin barrier abnormalities, although the consistency of TEWL measurements across studies is limited; succinate, mtDNA, and early Staphylococcus aureus colonization provide primarily mechanistic evidence, with unclear direct clinical predictive value; and TARC has limited predictive ability (AUC = 0.64) and is not a preferred marker for persistence prediction.

The biomarkers associated with disease persistence prediction in pediatric atopic dermatitis, including their detection time points, predictive values, and special considerations in children, are summarized in Table 2.

**Table 2:** Biomarkers associated with disease persistence prediction in pediatric atopic dermatitis.

Biomarker	Predictive value	Clinical status	Key limitation/Note	References
FLG loss-of-function mutations	Increases risk of atopic march and food allergy	Strongest genetic evidence	Limited sensitivity when used alone	(1, 7, 13, 33)
VEGF	Low levels predict persistence at 7 years of age (sensitivity 81.8%, specificity 82.4%); higher levels associated with remission by 7 years	Component of combined prediction model	Requires combination with clinical factors (SCORAD at 3 years, trigger factors) for comprehensive assessment	(34)
S100A8/9	Associated with persistent AD at 6–12 months of age	Population-specific	Only effective in FLG wild-type infants	(35)
Succinate	Mediates progression from AD to intestinal inflammation	Mechanistic evidence	Confirmed via tuft cell-ILC2 axis in animal models; direct clinical predictive value unclear	(1, 8)
mtDNA	Positively correlated with EASI and IgE levels	Mechanistic evidence	Participates in atopic march via STING pathway; direct clinical predictive value unclear	(1, 8, 36)
S.aureus colonization	Increase risk of peanut and egg allergy	Mechanistic evidence	Colonization precedes AD onset; direct clinical predictive value unclear	(27)
TARC	AUC = 0.64 for predicting AD onset within 12 months	Limited predictive ability	Inferior to skin lipid markers; not a preferred marker for persistence prediction	(10, 37)
NMF	Low levels increase risk of developing AD	Early barrier warning	Associated with FLG deficiency	(1)
TEWL	High levels predict AD in first year of life	Early barrier warning	Reflects skin barrier dysfunction; measurement consistency limited across studies	(1, 10)
Ceramides	C18/C20 ratio is among the best lipid biomarkers for predicting AD	Best predictive performance	Key feature of skin barrier dysfunction	(1, 10)
Phytosphingosine	Predictive accuracy 75.6%	Best predictive performance	Lipid biomarker	(10, 37)
Multi-lipid combination	Combined predictive accuracy 89.4%	Best predictive performance	Outperforms single lipid markers	(10, 37)

## 5 Non-invasive detection techniques

In recent years, a range of non-invasive or minimally invasive skin sampling techniques have shown promising potential for clinical application. While traditional blood samples and skin biopsies can provide rich diagnostic information, their invasive nature limits their application in large-scale clinical trials and pediatric research (1, 3). In the future, these techniques are expected to play a more important role in the early diagnosis and disease monitoring of pediatric AD.

### 5.1 Tape stripping

Tape stripping is a minimally invasive method capable of identifying distinct transcriptomic profiles characteristic of lesional and non-lesional AD skin (1). In clinical practice, 20 consecutive tape strippings do not cause bleeding or scarring, inducing only mild irritation (10). Studies have shown that tape stripping combined with high-throughput RNA sequencing may help reveal the pathogenesis of AD (38). Studies using this technique to detect stratum corneum IL-8 levels have shown significant reductions post-treatment, indicating that tape stripping can effectively monitor local treatment response (31).

### 5.2 Sebum RNA monitoring

The use of oil-blotting films to collect sebum for RNA analysis represents another non-invasive detection method (39). Using this technique, reduced expression of genes related to lipid metabolism and skin barrier function, along with elevated expression of genes associated with Th2, Th17, and Th22 immune responses, was detected in the sebum of infants with AD at 1 month of age (39). This technique can also predict the onset of AD at 2 months of age, holding significant value for understanding the molecular mechanisms of early-onset AD and facilitating early diagnosis (39).

### 5.3 Microneedle

The microneedle is a novel non-invasive detection technique. Hyaluronic acid microneedles (HA-MNs) with a length of 650*μ*m can penetrate the papillary dermis except on the palms and soles (40). Unlike tape stripping, HA-MNs can detect IL-4, IL-13, and IFN-*γ*. After successful AD treatment, monitoring with microneedles revealed decreased levels of IL-4 and IL-13, suggesting that microneedles can be used to monitor therapeutic progress (40).

## 6 Endotype classification

The high heterogeneity of AD dictates that different patients respond differently to the same treatment. Stratified therapy based on endotype classification is the key to achieving personalized treatment for AD (3, 41, 42).

Cluster analysis based on serum biomarkers can classify AD endotypes into four clusters: Cluster 1 is characterized by high levels of C-C chemokines and IL-1R1 predominance; Cluster 2 is characterized by a predominance of Th1-, Th2-, and epithelium-related chemokines; Cluster 3 is dominated by Th2, Th22, and PARC, associated with more severe disease; and Cluster 4 is a Th2- and eosinophil-low cluster with the mildest degree of inflammation (2).

Asian AD patients exhibit unique endotype characteristics, manifesting as upregulation of the Th2/Th17 axis and lower Th1 expression compared to European and American patients, displaying features similar to psoriasis (2, 4, 9).

### 6.1 Th2-type endotype

The Th2-type endotype is driven by key cytokines IL-4 and IL-13, and biologic agents targeting this pathway have been widely adopted in clinical practice.

#### 6.1.1 Dupilumab

Targets the IL-4 receptor alpha subunit, blocking both IL-4 and IL-13 signaling pathways (2, 41). Approved in 2022 for young children (6 months to 5 years), it is currently the only biologic approved for moderate-to-severe AD in children as young as 6 months (41). Real-world data from Napolitano et al. further supported its efficacy and safety in this age group, with a mean EASI reduction of 89.7% at week 52 and EASI-75/90 response rates of 79.5%/59.0% (5). Long-term extension data from Paller et al. (up to 104 weeks) confirmed sustained efficacy and a favorable safety profile, with no new safety signals identified (6).

#### 6.1.2 Tralokinumab and Lebrikizumab

Both are monoclonal antibodies targeting IL-13, blocking the IL-13 pathway (41). In adult studies, tralokinumab achieved IGA 0/1 in 15.8%–22.2% of patients and EASI-75 in 25.0%–33.2% at week 16 (43); in adolescents aged 12–17 years, tralokinumab achieved an EASI-75 response rate of 28% at week 16 (41). Lebrikizumab achieved IGA 0/1 in 33.2%–43.1% of adolescents aged ≥12 years at week 16, with significant improvement in EASI-75 (41). Both are approved for children aged 12 years and older (41). Notably, neither agent is currently approved for children under 12 years of age, and evidence for their use in younger pediatric populations remains insufficient.

#### 6.1.3 Nemolizumab

A humanized monoclonal antibody targeting the IL-31 receptor A, blocking the IL-31 pathway (41). A 2-year open-label extension study showed that after 104 weeks of treatment, IGA 0/1 was achieved in 62.6% of patients, EASI-75 in 88.2%, and pruritus improvement in 87.2% (44). However, this study enrolled patients aged ≥12 years, and data on the safety and efficacy of nemolizumab in children under 12 years of age remain insufficient.

#### 6.1.4 Mepolizumab and benralizumab

In adults, patients with the eosinophil-high subtype may benefit from anti-IL-5 therapy (mepolizumab) or anti-IL-5R*α* antibody (benralizumab) (3). However, current evidence for anti-IL-5 therapy in pediatric AD is extremely limited, and its potential value in the eosinophil-high subtype requires further validation.

#### 6.1.5 Omalizumab and ligelizumab

Both target the IgE pathway. By binding to free IgE, these agents reduce IgE-mediated mast cell and basophil activation, thereby attenuating downstream type 2 inflammatory responses (3). Theoretically, targeting the IgE pathway may be more applicable to extrinsic AD (characterized by elevated IgE), whereas its value in intrinsic AD (normal IgE) may be limited.

#### 6.1.6 Rocatinlimab

Targets the OX40 pathway. OX40 is a costimulatory receptor expressed on activated T cells, and blocking OX40-OX40L interaction can inhibit the expansion and survival of pathogenic memory T cells implicated in AD (3). Unlike biologics that directly neutralize cytokines, rocatinlimab acts by inhibiting the expansion and survival of pathogenic memory T cells. Theoretically, early intervention in AD may reduce the “memory” burden of pathogenic T cells, thereby influencing the long-term disease course—a strategy that targets a more upstream pathway than simple cytokine blockade. However, this mechanism may also require a longer time for clinical efficacy to fully manifest and may carry broader immunosuppressive effects. The practical implications of these potential benefits and risks in pediatric AD warrant further investigation.

### 6.2 Th17/Th22-type endotype

The Th17/Th22-type endotype is more common in Asian AD and intrinsic AD, driven by key cytokines IL-17, IL-22, and IL-23 (9).

#### 6.2.1 Secukinumab and risankizumab

IL-17 and IL-23 are potential therapeutic targets for non-type 2-dominant endotypes, corresponding to the anti-IL-17 agent secukinumab and the anti-IL-23 agent risankizumab (3). However, current evidence does not support the efficacy of IL-17 inhibition in AD, and further studies are needed to clarify the role of these pathways.

#### 6.2.2 Fezakinumab

Fezakinumab can downregulate Th1, Th2, Th17, and Th22-related immune pathways in patients with high serum IL-22 levels, demonstrating safety and efficacy in severe AD patients (2).

Notably, the Th17/Th22 endotype is more common in Asian patients with AD, suggesting that therapeutic strategies targeting IL-17/IL-23 may theoretically hold particular value for this population. However, current evidence does not support the efficacy of IL-17 inhibition in AD (3), which may reflect the complexity of AD pathogenesis—even when a Th17-dominant endotype is present, blocking IL-17 alone may be insufficient to adequately control the disease. In contrast, fezakinumab, which targets IL-22, has shown some efficacy in severe AD patients (2), suggesting that IL-22 may be a more relevant target closer to the core pathology of AD.

### 6.3 Mixed/other type

The Mixed/Other category involves the simultaneous activation of multiple inflammatory pathways or distinct mechanisms, requiring agents with broad-spectrum or alternative activities. JAK inhibitors target the JAK/STAT pathway and can simultaneously downregulate multiple inflammatory pathways in AD (2, 41), whereas PDE4 inhibitors act by increasing intracellular cAMP levels and reducing pro-inflammatory mediator transcription (2). These two classes represent distinct mechanistic approaches within the Mixed/Other category.

#### 6.3.1 Abrocitinib (JAK1 inhibitor)

Phase III trials in adolescents aged 12 years and older with moderate-to-severe AD showed that IGA 0/1 and EASI-75 response rates in the 100 mg and 200 mg groups were higher than those in the placebo group (41).

#### 6.3.2 Baricitinib (JAK1/2 inhibitor)

The 4 mg dose resulted in a higher proportion of patients achieving EASI-75 at week 16, with significant reductions in pruritus and sleep disturbance. It is approved for moderate-to-severe AD in patients 2 years and older (41).

#### 6.3.3 Upadacitinib (JAK1 inhibitor)

The 15 mg and 30 mg doses were significantly superior to placebo in achieving EASI-75, vIGA-AD 0/1, EASI-90, and pruritus relief, and it is approved for children aged 12 years and older (41).

#### 6.3.4 Topical JAK inhibitors

Delgocitinib ointment applied in children aged 2–15 years showed that, compared with the placebo group, the 0.25% and 0.5% delgocitinib groups had significantly reduced EASI and IGA scores (41).

#### 6.3.5 Phosphodiesterase 4 (PDE4) inhibitors

Crisaborole (a PDE4 inhibitor) previously received European marketing authorization for mild-to-moderate AD in patients 2 years and older (later withdrawn in January 2022 for commercial reasons); difamilast (another PDE4 inhibitor) has shown positive results in phase II trials in children and adults (2).

The targeted therapeutic agents for atopic dermatitis, classified by endotype and molecular pathway, are summarized in Table 3.

**Table 3:** Endotype-based classification of targeted therapeutic agents for AD.

Endotype	Pathway	Agents	Pediatric approval/Age indication
Th2 type	IL-4/IL-13 pathway (shared)	Dupilumab	≥6 months (FDA, EMA, NMPA)
Th2 type	IL-13 pathway	Tralokinumab, Lebrikizumab	≥12 years (FDA, EMA); not approved for pediatric AD (NMPA)
Th2 type	IL-31 pathway	Nemolizumab	≥12 years (FDA, EMA); not approved for pediatric AD (NMPA)
Th2 type	IL-5 pathway	Mepolizumab, Benralizumab	Not approved for pediatric AD (FDA, EMA, NMPA)
Th2 type	IgE pathway	Omalizumab, Ligelizumab	Omalizumab: Not approved for pediatric AD (FDA, EMA, NMPA); Ligelizumab: Not yet approved in pediatrics for any allergic indication
Th2 type	OX40 pathway	Rocatinlimab	Insufficient pediatric AD clinical evidence, not approved for pediatric AD (FDA, EMA, NMPA)
Th17/Th22 type	IL-17 pathway	Secukinumab	Not approved for pediatric AD (FDA, EMA, NMPA)
Th17/Th22 type	IL-22 pathway	Fezakinumab	Insufficient pediatric AD clinical evidence, not approved for pediatric AD (FDA, EMA, NMPA)
Th17/Th22 type	IL-23 pathway	Risankizumab	Not approved for pediatric AD (FDA, EMA, NMPA)
Mixed/Other type	JAK1 inhibitor	Abrocitinib, Upadacitinib	≥12 years (FDA, EMA, NMPA)
Mixed/Other type	JAK1/2 inhibitor	Baricitinib	≥2 years (EMA); not approved for pediatric AD (FDA, NMPA)
Mixed/Other type	Pan-JAK inhibitor	Delgocitinib	≥2–15 years (Japan approval only; no FDA/EMA/NMPA pediatric AD approval)
Mixed/Other type	PDE4 inhibitor	Crisaborole, Difamilast	Crisaborole: ≥3 months (FDA, NMPA); EMA marketing authorization withdrawn Jan 2022 (originally approved for ≥2 years, never marketed in EU); Difamilast: ≥2 years (FDA); not approved for pediatric AD (EMA, NMPA)

Notes for“Pediatric Approval/Age Indication”: Approval data retrieved from official FDA, EMA and NMPA documents (cut-off: Aug 5, 2026).

U.S. Food and Drug Administration (FDA), European Medicines Agency (EMA), National Medical Products Administration (NMPA).

## 7 Discussion

This review systematically summarizes the current landscape of biomarkers for pediatric atopic dermatitis (AD), focusing on three key clinical applications: severity assessment, persistence prediction, and non-invasive monitoring. Several important findings, controversies, and research gaps emerge from this synthesis.

### 7.1 Key findings and clinical implications

In terms of severity assessment, the evidence reviewed in this study indicates that biomarkers differ substantially in their strength of evidence. TARC/CCL17 has been consistently validated as the most reliable severity marker across multiple studies, with pooled correlation coefficients of 0.6 in longitudinal randomized trials and 0.64 in cross-sectional studies. Periostin has shown correlations with EASI and pruritus scores and demonstrated potential in predicting response to IL-13 inhibitors, but its independent predictive value requires further validation. Eosinophil counts and total IgE have clinical utility in specific contexts (e.g., distinguishing extrinsic from intrinsic AD), but eosinophils have limited sensitivity in intrinsic AD; while IgE, in addition to this issue, also has poor specificity and weak correlation with severity. Emerging markers such as CCL26, SCCA2, and LDH show promise but require large-scale validation.

For persistence prediction, the evidence reviewed in this study indicates that biomarkers differ substantially in their predictive value, target populations, and strength of evidence. FLG loss-of-function mutations have the strongest genetic evidence, increasing the risk of multiple comorbidities by 2- to 3-fold and being closely associated with asthma, food allergy, and the atopic march, although their sensitivity is limited when used alone. VEGF is a component of a combined prediction model and requires combination with clinical factors (SCORAD at 3 years of age, trigger factors) for comprehensive assessment, whereas S100A8/A9 is only effective in FLG wild-type infants. Skin lipid markers (e.g., ceramide ratios, phytosphingosine) demonstrate the best predictive performance, with a combination of five lipid ratios achieving 89.4% accuracy. Barrier function measures (NMF, TEWL) and barrier composition markers (ceramides, phytosphingosine) can help identify early skin barrier abnormalities, although the consistency of TEWL measurements across studies is limited. Succinate, mtDNA, and early Staphylococcus aureus colonization provide primarily mechanistic evidence, with unclear direct clinical predictive value. TARC has limited predictive ability (AUC = 0.64) and is not a preferred marker for persistence prediction.

### 7.2 Controversies and debates

Several controversies in pediatric AD biomarker research warrant explicit discussion. First, while Th2-dominant inflammation is well-established in both children and adults, the relative contributions of Th17 and Th22 pathways remain debated. Current evidence suggests Th17 and Th9 predominance in pediatric patients vs. Th22 predominance in adults. However, whether this represents true age-related immunological maturation or reflects differences in disease chronicity requires longitudinal studies that track endotype evolution from infancy through adolescence.

Second, the role of IL-36 as a severity biomarker remains contested. Although multiple studies have reported elevated mRNA expression of IL-36*α* and IL-36*γ* in AD lesional skin, protein-level evidence has been inconsistent, with some studies failing to detect corresponding protein expression in the same tissue type. This mRNA-protein discordance may reflect the complexity of IL-36 expression regulation—its protein levels are influenced by post-transcriptional mechanisms, translational efficiency, and protease activity; methodological differences across studies (RNA sequencing/RT-PCR vs. immunohistochemistry) and variations in sample sources may also contribute to the discrepant results. This inconsistency suggests that IL-36 expression in AD is not yet well-defined and currently insufficient to serve as a preferred severity biomarker.

Third, the clinical utility of total IgE remains limited despite its widespread use. Its weak and inconsistent correlation with disease severity, coupled with poor specificity (it is also elevated in asthma, allergic rhinitis, and other atopic conditions), questions its value as a standalone marker. However, combining IgE with eosinophil counts successfully distinguishes extrinsic from intrinsic AD, suggesting that its optimal role is as part of multi-marker panels rather than as a solitary indicator.

Fourth, the Asian AD endotype—characterized by Th17/Th22 upregulation with lower Th1 expression compared to European/American patients—challenges a one-size-fits-all biomarker approach. It remains unclear whether Asian children exhibit this mixed phenotype from disease onset or develop it over time. Furthermore, most validation studies have been conducted in Western populations, limiting the generalizability of current biomarker cut-offs to Asian pediatric cohorts.

Given that Asian AD patients exhibit a unique endotype characterized by Th17/Th22 upregulation and lower Th1 expression compared to European/American patients (2, 4, 9), a theoretical question arises: whether this altered immune profile might influence the clinical response to IL-4/IL-13 blockade in Asian children. While head-to-head trials comparing dupilumab efficacy across ethnic groups are lacking, the biological premise suggests that Th17/Th22-dominant inflammation may be less dependent on IL-4/IL-13 signaling, potentially leading to differential treatment responses. However, the substantial efficacy observed in global trials does not support a clinically meaningful resistance to dupilumab in Asian populations. Nevertheless, the proportion of Asian patients in these trials was relatively small, and dedicated subgroup analyses are warranted to clarify whether endotype-driven differences in treatment response exist. Future studies incorporating baseline biomarker profiling (e.g., IL-17/IL-22 levels) may help identify which Asian AD patients are most likely to benefit from IL-4/IL-13 blockade vs. those who might require additional or alternative targeting of Th17/Th22 pathways.

### 7.3 Research gaps and future directions

Several critical gaps impede clinical translation. Most biomarkers lack pediatric-specific reference intervals, with existing thresholds derived from adult studies. Given the well-documented age-related differences in immune profiles (Th17 and Th9 predominance in children vs. Th22 in adults), adult-derived cut-offs may misclassify disease severity in children.

Additionally, most studies are cross-sectional with modest sample sizes, which limits the assessment of biomarkers' predictive value for long-term outcomes such as disease persistence, progression of the atopic march, or durability of treatment response. Longitudinal birth cohort studies with serial biomarker measurements are urgently needed.

Future efforts should prioritize: (1) Establishing pediatric reference intervals through large-scale, multi-center prospective studies stratified by age (infants, toddlers, school-age children, adolescents). Specifically, in future studies, ROC curve analyses stratified by age groups (e.g., 0–2 years, 2–6 years, 6–12 years) could be performed using existing birth cohorts (e.g., the Copenhagen Infant Skin Cohort) or prospective study data to determine optimal cut-off values for each age group. For example, the Youden index could be applied to candidate biomarkers such as TARC/CCL17 to identify cut-offs with both high sensitivity and specificity, and their predictive performance could be further validated in prospective cohorts; (2) Developing multi-marker panels incorporating TARC, eosinophils, CCL26, and skin barrier markers to improve predictive accuracy beyond any single biomarker; (3) Validating machine learning models that integrate clinical, genetic, and biomarker data for endotype classification and treatment response prediction; (4) Standardizing non-invasive sampling protocols (tape stripping, sebum RNA, microneedles) to facilitate routine clinical implementation.

## 8 Conclusion

In summary, significant progress has been made in the identification of biomarkers for pediatric AD, particularly in severity assessment and persistence prediction. Targeted therapies—including biologics targeting Th2, Th17/Th22, and mixed endotypes, as well as JAK inhibitors—have increasingly entered pediatric clinical practice, and non-invasive techniques (e.g., tape stripping, sebum RNA analysis, microneedles) have provided new tools for early diagnosis and dynamic monitoring in children. However, most biomarkers still lack pediatric-specific reference intervals and large-scale validation, and long-term safety and efficacy data for targeted therapies in young children remain insufficient. Future efforts should focus on establishing multi-center prospective cohorts, developing multi-marker panels, and standardizing non-invasive techniques to facilitate clinical translation, ultimately enabling early risk stratification and personalized management of pediatric AD.
